# User-centered design of a web-based self-management site for individuals with type 2 diabetes – providing a sense of control and community

**DOI:** 10.1186/1472-6947-14-60

**Published:** 2014-07-23

**Authors:** Catherine H Yu, Janet A Parsons, Susan Hall, David Newton, Aleksandra Jovicic, Danielle Lottridge, Baiju R Shah, Sharon E Straus

**Affiliations:** 1 Li Ka Shing Knowledge Institute, St. Michael’s Hospital, 30 Bond Street, Toronto, ON M5B 1 W8, Canada; 2 Department of Medicine, St. Michael’s Hospital, University of Toronto, Toronto, Canada; 3 Dhalla Lana School of Public Health, University of Toronto, Toronto, Canada; 4 Applied Health Research Centre, Li Ka Shing Knowledge Institute, St. Michael’s Hospital, 30 Bond Street, Toronto, Canada; 5 Department of Physical Therapy and Graduate Department of Rehabilitation Science, University of Toronto, Toronto, Canada; 6 Department of Mechanical and Industrial Engineering, University of Toronto, Toronto, Canada; 7 Institute for Health Policy, Management and Evaluation, University of Toronto, Toronto, Canada; 8 Sunnybrook Research Institute, Sunnybrook Health Sciences Centre, Toronto, Canada; 9 Institute for Clinical Evaluative Sciences, Toronto, Ontario, Canada

**Keywords:** Diabetes mellitus, Self-efficacy, Intervention development, User-Computer Interface, Qualitative methods

## Abstract

**Background:**

To design and test a web-based self-management tool for patients with type 2 diabetes for its usability and feasibility.

**Methods:**

An evidence-based, theory-driven website was created for patients with type 2 diabetes. Twenty-three patients with type 2 diabetes aged ≥ 25 years were recruited from 2 diabetes care centers in Toronto, Canada. We employed focus group methodology to assess acceptability, sustainability, strengths and weaknesses of the self-management website. Based on these results, revisions were made to the website. Three cycles of individual usability testing sessions using cognitive task analysis were conducted with patients with type 2 diabetes. Revisions to the website were made based on results from this testing.

**Results:**

We identified five themes concerning participants’ experiences of health care and related unmet needs: 1) *Desire for information* and for greater access to timely and personalized care to gain a sense of control of their disease; 2) *Desire for community* (sharing experiences with others) to fulfill practical and emotional needs; 3) *Potential roles of an online self-management website* in self-empowerment, behavior change, self-management and health care delivery; 4) *Importance of a patient-centered perspective* in presenting content (e.g. common assumptions, medical nomenclature, language, messaging, sociocultural context); 5) *Barriers and facilitators to use* of a self-management website (including perceived relevance of content, incorporation into usual routine, availability for goal-directed use, usability issues).

**Conclusions:**

Participants outlined a series of unmet health care needs, and stated that they wanted timely access to tailored knowledge about their condition, mechanisms to control and track their disease, and opportunities to share experiences with other patients. These findings have implications for patients with type 2 diabetes of diverse ages, socioeconomic backgrounds, and disease severity, as well as to the design of other computer-based resources for chronic disease management.

## Background

Clinical care gaps are common in diabetes care. In the United States, for example, in 2010, the Behavioral Risk Factor Surveillance System estimates that only 68% of patients with type 1 or type 2 diabetes had an A1c measured at least twice in the past year, and only 63% and 68% had retinal and foot examinations, respectively, in the past year [1]. This is despite recommendations from the American Diabetes Association that the former be measured at least 2 to 4 times per year, and that patients undergo annual retinal and foot exams [2]. Given that patients provide the majority of their own diabetes care [3], patient self-management, where patients take responsibility of their own behavior and well-being, is increasingly recognized as an important strategy with which to potentially improve quality of care [4]. For example, daily self-management tasks of a typical person with diabetes include self-monitoring of blood glucose and blood pressure, dietary modification (with consideration of carbohydrate quantity and quality, saturated fat intake, portion size, sodium and potassium content), engagement in physical activity and self-administration of antihyperglycemic, lipid-lowering, antihypertensive, antiplatelet and vasculo-protective medications [5]. However, participation in self-management education programs is low [6,7]. In addition, the effectiveness of existing behavioral interventions wanes over time [8], reducing the long-term impact of self-management interventions. Web-based interventions have the potential to bridge these gaps in diabetes care and self-management [9-11]. Effective education and self-management principles, such as cognitive, behavioral and social strategies including goal-setting, problem-solving and motivational techniques, have not been systematically incorporated into existing diabetes websites for patients and usability problems are common in websites, limiting the effectiveness and reach of these resources [12,13].

We sought to reduce the clinical care gap through the development and use of a web-based patient self-management intervention. During intervention development, use of theory-based strategies targeted to determinants of knowledge uptake is thought to increase the probability of successful implementation [14]. Eliciting user input and feedback in a systematic manner can be used to identify determinants of knowledge uptake and can facilitate development of a usable interface for the proposed web-based intervention [15]. In this paper, we describe our intervention development and refinement, as well as qualitative results from the initial phases of this multi-phased research project.

## Methods

**Figure 1:**
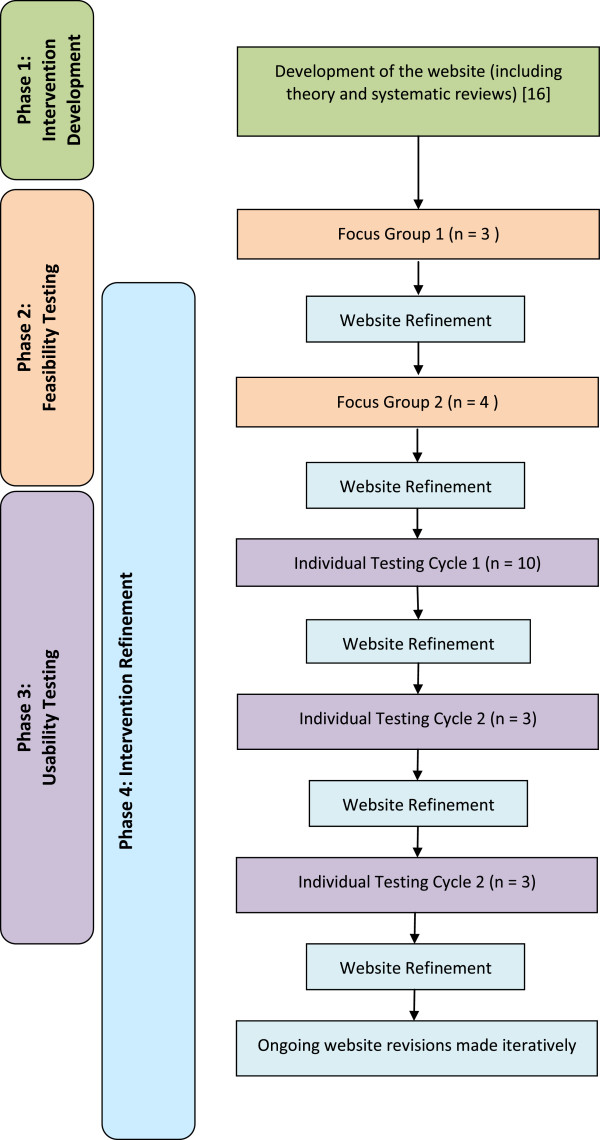
Study flow diagram.

Briefly, this investigation was part of a broader study to develop and evaluate a self-management website. The broader study consisted of five phases: Phase 1: Intervention development; Phase 2: Feasibility testing; Phase 3: Usability testing; Phase 4: Intervention refinement; and Phase 5: Intervention evaluation. The study protocol is described in detail elsewhere [16]. A mixed methods approach was adopted; quantitative and qualitative methods were used. This paper focuses specifically on the qualitative findings generated in Phases 1 through 4 (Figure 1).

### Phase 1: intervention development

**Figure 2:**
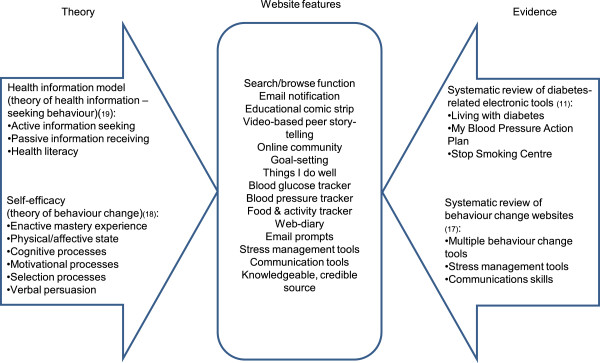
Evidence-based and theory-driven framework was used for intervention development.

**Figure 3:**
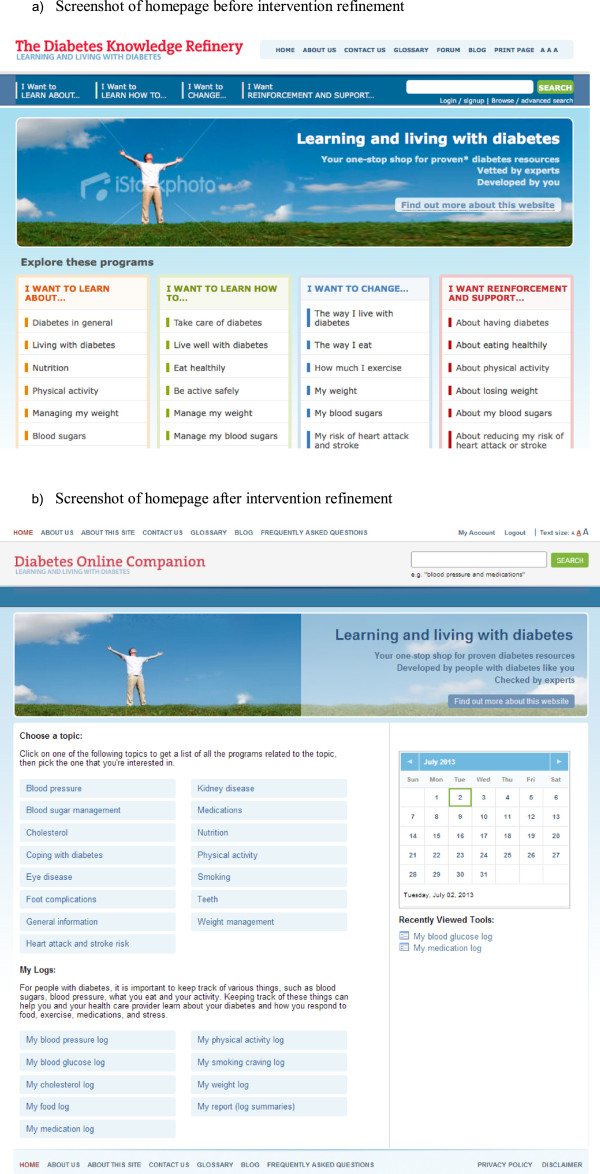
**Screenshots of homepage before and after intervention refinement. a** Screenshot of homepage before intervention refinement. **b** Screenshot of homepage after intervention refinement.

Our objective was to create an evidence-based, theory-informed self-contained website focused on facilitating the management of diabetes including optimising vascular risk factors. Figure 2 depicts our evidence-based and theory-driven framework that was used for intervention development. Details regarding systematic reviews of diabetes-related electronic tools [11] and behavior change websites [17], the theory of self-efficacy [18], the Health Information Model [19], as well as their application to our website development are described elsewhere [16]. We selected self-management tools known to be effective, relevant and usable [11]. Though multiple theories could guide this work, we selected self-efficacy, a theory that has not only been validated in predicting and promoting patient behavior change but has also been demonstrated to improve clinical outcomes in diabetes care [20-27]. Guided by this theoretical framework, sources and mediators of self-efficacy were integrated into website format and tool selection. Feedback, goal-setting, peer story-telling, and monitoring tools were incorporated. In order to complement patient health-information-seeking behavior [19] we sent automated emails with selected content (such as tailored reminders to complete a self-management log, or new content on the website), optimized search algorithms to enable self-directed information retrieval, and included tools to facilitate communication with health care providers (HCPs). Our overarching design goal was to tailor the website to patient characteristics, include their age, computer familiarity, behavioral characteristics (stage of change, self-efficacy, self-care) and stage of disease. To achieve this, we created a combination of tag-based and hierarchical organization; in other words, we presented a combination of “look around yourself” and guided “step by step” approaches. We included features such as definitions under a mouse hover mode, and links to additional definitions such as levels of evidence. We clustered our content in the areas of knowledge, behavior change, skill development and reinforcing/supporting resources. Throughout the website, we chose labels and titles carefully so that it would be clear and understandable. Figure 3a depicts how the system looked before any user evaluation was done. The iterative design began in this phase where the designers met with human factors specialists and content experts to iteratively refine the website based on discussion of user needs and tasks.

### Phase 2: feasibility testing

We conducted a preliminary evaluation of our proposed intervention in order to determine whether an investment into a complete intervention was justified [28]. Specifically, we employed focus group interviews each lasting 90 minutes, to identify general themes regarding acceptability, usability, sustainability, as well as strengths and weaknesses of the website. We selected focus groups because a critical benefit of eliciting information through focus groups instead of multiple one-on-one interviews is that the group discussion can reveal areas of consensus and disagreements in the topic domain [29]. Participants viewed the website on individual computers and were asked to complete a task simulating real clinical usage, specifically, to determine their risk of heart disease and strategies by which they can reduce this risk.

Participants: A purposive sampling strategy was used to ensure sample heterogeneity [30], in order to capture the perspectives of participants with varied experiences, including those of different ages (≤60 years old, >60 years old), gender, duration of diabetes (≤5 years, > 5 years), educational attainment (no post-secondary, post-secondary) and annual income (≤$40000, >$40000). Participants (aged greater than 25 years) were recruited from diabetes care centers at two academic health science centers in Toronto, Canada. After the attending physician made initial contact with possible participants in the course of a regular clinic appointment, the research coordinator then approached the patient in person after the clinic visit. After verbal explanation, the research coordinator provided the subject with written consent forms. Data collection: Participants viewed the website and explored its content during the focus groups led by SH and DL. We elicited comments on website content and format and factors that they felt would determine its use. A semi-structured interview guide was developed by team members with knowledge translation and qualitative research expertise, and included questions about barriers and facilitators to website use, the role of social networking, and comfort with entering personal information online. This interview guide (Additional file 1: S1) was refined iteratively based on analysis of preceding focus group transcripts. All focus group interviews were audio recorded and field notes kept.

Data analysis: Audio tapes were transcribed verbatim [31] and coded using a descriptive content analytic approach [32,33]. Analysis began with the completion of the first focus group and results were used to modify the interview guide. Transcripts were reviewed independently by three team members with experience in qualitative research methods; consensus on coding was reached through comparison, discussion, and agreement among these three reviewers [34]. We employed an inductive emergent approach and used multiple coders [35], in order to discuss the emerging analytic framework and to explore alternative explanations of the data and address the potential for multiple interpretations.

### Phase 3: usability testing

After refinements were made to the self-management website based on the results of the feasibility testing, we conducted individual usability testing sessions, each lasting 60 minutes, using cognitive task analysis [36] and in-depth interviews to drill down to specific use cases of the website. Cognitive task analysis is the characterization of the decision making and reasoning skills, and information processing needs of subjects as they perform activities/tasks involving the processing of complex information [36]. Cognitive task analysis was selected as the most appropriate tool for usability testing as it provides a first-hand look at how representative users interact with the product so they can determine what needs to be addressed [36]. Real users often do and say unpredictable things that expert reviewers cannot anticipate, in particular with populations with unique characteristics or experiences such as living with a chronic illness. We used the think-aloud method, giving our participants the following instructions and example: “As I mentioned before, while you are using the website, we are interested to know what you’re thinking, and we use a method called ‘Think Aloud’. What we mean by think aloud is that we would like for you say everything that you’re thinking. In other words, you will be constantly talking, telling us what you are thinking inside your head. For example, if I were to think aloud while trying to find the sixth letter of the alphabet, I would say all the letters and use my fingers to count a b c d e f there I found it”. In accordance with the principles of iterative design [37], the usability testing was iterative: the first usability session was conducted with a group of representative participants; the usability problems identified were fixed through redesign; a second session was conducted with a new group of participants, and any remaining usability issues were fixed. According to the principles of iterative design, this process was repeated until all critical usability issues were addressed [37]. In each round of usability testing, participants were asked to complete several tasks which reflect questions that may arise in self-management of diabetes. In the first iteration of the usability testing, participants were asked to complete the following: 1) determine what blood pressure is, its impact, strategies to control it and how to record it; and 2) determine whether leg pain was related to the risk of heart disease or stroke. In the second iteration of usability testing (for which we completed 2 cycles of iterative design), participants were asked to complete 5 different tasks, selected to assess the full functionality of different website tools: 1) subjectively assess reliability of website information; 2) determine what blood pressure is, its impact, strategies to control it and how to record it; 3) record their medications; 4) search for a comic (an animated graphic story describing the struggles of a person living with diabetes); and 5) interpret the comic (Additional file 1: S2).

Participants: A consecutive sample of 16 participants (n = 10 in first iteration, n = 6 in second iteration) with type 2 diabetes aged ≥25 years were recruited, as detailed above, from diabetes care centers at two academic health science centers in Toronto, Canada. Up to 80% of usability issues can be identified with 5 to 8 participants [36,38].

Data collection: We collected data on paths users took to accomplish tasks, usability problems encountered, when and where they became confused or frustrated using the website, whether they completed the task, and overall impressions of the website through questions such as “What did you like about the web site?”, “What did you not like?”, “Do you think any information is missing?”. We opted not to collect data regarding time on task due to the subjective nature of the majority of the tasks. A consultant with expertise in health informatics and human factors engineering (AJ, DL) conducted each session. All individual usability testing sessions were video recorded and field notes kept. These sources of data collection allowed us to identify the path that users took to complete the various tasks.

Data analysis: The approach to data analysis for usability testing was identical to that described under feasibility testing, including iterative interview guide development, with the additional use of visual data to analyze paths taken by users to complete the tasks given to them. We triangulated datasets from both phase 2 and 3 to develop themes. Triangulation consisted of: 1) examining the usability data through the lens of “perceived value and role of website to users”; 2) corroborating feasibility findings with usability findings; and 3) interrogating how users’ expectations impact usability and design [39].

### Phase 4: intervention refinement

Based on data from usability and feasibility testing, refinements were made iteratively to the website via ongoing discussion with the research and development team (CY, SH, DN, AJ, DL, SES), described below [37].

The study was approved by the Research Ethics Boards of St. Michael’s Hospital and Sunnybrook Health Sciences Centre.

## Results

### Phases 2 and 3: themes identified from feasibility and usability testing

Two focus groups involving seven patients with type 2 diabetes (3 and 4 participants in groups 1 and 2 respectively) were conducted during the feasibility testing. The first cycle of usability testing included 10 participants, followed by two additional cycles, each with 3 participants (total n = 16 for Phase 3). Thus, in total, we conducted 5 cycles of feedback and redesign. Because analysis of both datasets identified very similar themes, we report the results from Phases 2 and 3 together. Demographic characteristics for the entire sample of 23 patients are listed in Table 1. Five themes were identified: 1) *Desire for information* and for greater access to timely and personalized care to gain a sense of control of their disease; 2) *Desire for community* (sharing experiences with others) to fulfill practical and emotional needs; 3) *Potential roles of an online self-management website* in self-empowerment, behavior change, self-management and health care delivery; 4) *Importance of a patient-centered perspective* in presenting content (e.g. common assumptions, medical nomenclature, language, messaging, sociocultural context); 5) *Barriers and facilitators to use* of a self-management website (including perceived relevance of content, incorporation into usual routine, availability for goal-directed use, usability issues). Representative quotes for each theme are reported in Table 2.

**Table 1:** Characteristics of focus group and usability participants

		**Focus group (n = 7)**	**Usability (n = 16)**
Gender	Male	2 (29%)	10 (62.5%)
	Female	5 (71%)	6 (37.5%)
Age	20 to 29 years old	0	2 (12.5%)
	40 to 59 years old	3 (43%)	4 (25%)
	60 to 79 years old	4 (57%)	10 (62.5%)
Insulin use	Yes	5 (71%)	7 (44%)
	No	2 (29%)	9 (56%)
Duration of diabetes	< 5 years	2 (29%)	4 (25%)
	5 to 14 years	4 (57%)	6 (37.5%)
	>15 years	1 (14%)	6 (37.5%)
Cardiac risk factors	Hypertension	4 (57%)	11(69%)
	Dyslipidemia	3 (43%)	8 (50%)
	Smoker	0	1 (6%)
Education	High school	2 (29%)	2 (12.5%)
	College or University	5 (71%)	14 (87.5%)
Annual income	< $15 000	2 (29%)	4 (25%)
	$15 000 to $29 999	0	1 (6%)
	$30 000 to $59 999	3 (43%)	3 (19%)
	$60 000 to $89 999	1 (14%)	5 (31%)
	> $90 000	1 (14%)	3 (19%)
Comfort with computer use	Very comfortable	3 (43%)	11 (69%)
	Somewhat comfortable	0	4 (25%)
	Neutral	0	1 (6%)
	Somewhat uncomfortable	1 (14%)	0
	Did not respond	3 (43%)	0

**Table 2:** Themes identified and representative quotes from feasibility focus groups and usability testing

**Themes**	**Representative quotes**
**1) Desire for information**	*“I want to be able to put maybe my question on here and then maybe have a doctor or nurse come in and give me sort of an immediate answer, because I’m not going to see my specialist for three months. … People like … something that’s immediate.”* [2A09]
**2) Desire for community**	*a) “Recently, I’ve been feeling like I need to talk to someone because some things are happening now, like I’m experiencing tingling and stuff like that. This is all new to me, so I’m starting to kind of freak out about it. Like, I’ve seen people about it, but to be able to say, ok, what do you guys experience? To have that comfort level, as a support, even if it’s online, so that would bring me back to society, to have that connection to someone, others that are going through the same thing. You know, friends and family just don’t fully really understand.”* [1A12]
	*b) “Sometimes you want to have a really quick answer or something and you’re trying to look for the answer, but you can only talk to certain people at certain times. If you could just type in a question and maybe other people, other diabetics, might be able to answer the question … To be able to personalize I think … would be great.”* [1A12]
	*c) “There are people who are sensitive… They have diabetes but they don’t want to tell people… They don’t want to tell friends, or family.”* [2B02]
	*d) “But people treat you differently. [Even though] it’s ok to have a piece of cake now and then, but [if] people hear you have diabetes, [they say] “Should you be touching that!?” All of a sudden, people are focusing in on you and you are just trying to be part of the crowd and they’re just all of sudden coming at you.”* [2A09]
**3) Potential roles of an online self-management website**	
*○ Motivate for behavior change*	*a) “But when I saw the woman who essentially came out with the do’s and don’ts of the disease, that kind of enlightened me, it opened my mind, it made me hopeful. It made me think: “Well, there is an alternative: I should take care of myself, I should recognize the issues, I should take care of my feet which means cleaning them more often, I should see a foot doctor who addresses the calluses”. So I think that the videos have enlightened me to a point where I have realized the detriment of the disease, where I realized the precautionary measures I should keep in mind in order to not to get to that stage.”* [3A21]
*○ Facilitate self-monitoring and self-management tasks*	*b) “And what I would do now is go back and take a look to see how to interpret that blood pressure: What does it mean, should I change something?”* [3B56]
	*c) “And complete this, rather than developing your own form which I do now, go on the computer and try to develop a form, this is so much easier and this would remind me that I didn’t take it.”* [3B51]
	*d) “I think you have to be anal retentive to do this quite frankly.”* [3B17]
*○ An adjunct to care between visits to HCPs*	*e) “Doctors, practitioners don’t have time. They’ve got their waiting rooms full and they have got so much allotted time for each one, so they just deal with it immediately, and there is no time for questions, no time for research, there’s no time for anything.”* [2A09]
	*f) “I would probably go to it because as a diabetic you don’t want to go all the way to a doctor to ask the questions, and knowing that this site is monitored and put together by health professionals, it would be nice to go and get an answer when you need it, instead of saying, oh when I see the doctor the next time…”* [3B17]
*○ Facilitate interaction with HCPs*	*g) “I don’t know if it’s there in this program where I can record everyday and then make a print out to take to the specialist or the doctor to show them what’s happening.”* [3A19]
**4) Importance of a patient-centered perspective**	*a)* One participant commented regarding a video that graphically depicted the consequences of poor dental hygiene in diabetes, which concluded with an upbeat message, that: *“And it’s not negative in the sense that the information it’s trying to transmit is negative; it’s negative looking at the results of not caring. That’s the negativity feeling that I’m having. It turns your stomach, you know. But the information itself is positive. So here I have a sort of mixed bag of feelings: really the negativity of looking at uncared teeth, and the positiveness of getting help to ensure that it doesn’t get to that point.”* [3A21]
	b) *“People are going to come from different points of view, different education levels and most importantly, different cultural backgrounds, and right now, my first reaction is to comment and say this is great for anyone that was raised in the Western society.”* [3B12]
**5) Barriers and facilitators to use**	
*○***Barriers**	
*○ Perceived lack of relevance:*	a) “*Always seemed to me that they were related more to people that are sedentary.”* [3B56] (when commenting on current publicly available websites)
*○ Not part of usual routine:*	*b) “You know, personally I wouldn’t use it. ‘Cause… maybe it’s because of my routine. Again, I’m very focused in the morning: I get up, this is what I’ve got to do, I’ve got my stuff right handy next to the bed, so I take it go on to the washroom and power down the pills, and then have my breakfast. It’s my routine.”* [3B56]
	*c) “I’m not every day in front of a computer so, I usually go once in the morning and once before bed to check what I’ve got on and through the day I don’t bother.”* [3A02]
*○***Facilitators**	
*○ Availability for goal-directed use:*	*d) “Like the thing about the nerves it bothered me, you know, when I heard that you can get an amputation as a result so I went in and did a whole read up on nerves and how to take care of it. But that’s just me. That’s what I like to do on my spare time. And something like this I would be on it all the time…enjoying myself and have a few there that all the time.”* [3A21]

In general, participants thought that the website was clear, comprehensive yet concise, relevant, and approachable:

*“And it seems to be friendly enough. It doesn’t slap you around and say you’ve got to do it this way or else. It just lets you go through that.”* [3B56]

#### Desire for information and for greater access to timely and personalized care

Interacting with the website elicited reflections from participants on their experiences with diabetes. In their accounts, they struggled against a sense of futility, against a sense of loss of control of their condition. For example, in reference to a webpage which listed potential complications of diabetes, one participant commented: *“I just find that all of these complications are so predestined, that no matter what you do, you are going to get these”* [2B01]. However, some participants countered this sense of futility, portraying diabetes instead as an entity to be controlled, and persevered with attempts to control their diet, blood glucose and blood pressure, to *“feel more in command of their life”* [3B17]. Another participant framed her approach to her diabetes as something for her to control: *“If my (blood sugar) reading is high, what do I have to do to bring it down? What do I have to do to normalize it? And I would look for (a) way, something that I can control”* [3A21].

When interacting with the website during both the feasibility and usability testing, participants said they sought health information to acquire greater knowledge about diabetes and to gain a sense of ‘control’ over their disease and its impact on their lives. They spoke of the need for timely and personalized information (Table 2; Quote 1). Participants indicated that obtaining immediate information fostered a sense of ‘control’ over that aspect of their life. They indicated that an online system could potentially fulfill this need.

While all the participants felt that access to timely and relevant information was important, they also noted that the amount of information and its presentation are crucial to their comfort level using the website. Participants’ accounts reflected a tension between a desire for enough information and desire to not be overloaded with information. On the one hand, participants expressed that they needed detailed information in order to manage particular features of the disease as they arise:

*“More information in there that would be more relevant to you. When these things happen to you, you become much stressed and you’re looking for answers. So it would be more helpful if you had a more detailed description of what causes the leg pain.”* [3A29]

On the other hand, too much information can lead to participants feeling overwhelmed:

*“Yes, I need to know about those, but I don’t want it to be thrown in my face all at once, it’s like getting hit by a car.”* [2A09]

The language this participant uses speaks to the degree to which information overload can impact patients; this participant uses very strong imagery to express his/her almost visceral responses provoked by this perception of information overload. The perception of excessive information had the potential to drive people away from the website thus reducing website usage unless they were able to tailor the amount of information received to their individual needs. This finding suggests that the ideal website should present the user first with a concise overview of the website content, which the user could then select for further detail and tailor down to his or her needs.

Participants indicated that they were seeking ways to interact with the website that would allow them to sample given information on an ‘as needed’ basis, without having to wade through a lot of extraneous information not perceived to be particularly relevant to their specific concern.

*“I guess because there is a lot of information there. I feel like it’s taking me a bit of time to get through what the lists are; it’s just that I’ve got to find it. Once you know the flow, I can go back in.”* [3B56]

The balance of amount and type of information appeared to be dependent on their level of interest or need for a particular topic at a given time. We addressed this need for “balance” by revising website layout, organization and navigation to permit an individualized approach where they could seek relevant information on an “as needed” basis. Participants wanted information about medications (including their purpose and mechanisms of action), the role of various health care professionals, new breakthroughs in diabetes and diabetes research, and the role of physical activity in diabetes management and prevention. Additional content requested by participants is listed in Additional file 1: S3.

Participants seemed to want to combat a sense of loss of control that resulted from having diabetes. One mechanism by which they said they could regain a sense of control was by being able to access “just enough” of the “right” information “now”. Thus, this concept of “keeping the user in control” was identified as a crucial consideration in ensuring user engagement with the website.

#### Desire for community (sharing experiences with others) to fulfill practical and emotional needs

Participants stated they wanted access to an online community to fulfill practical and emotional needs that arose around managing a chronic disease. For example, they wanted to share their experiences, assuage insecurities and fears, and obtain social and emotional support. Participants wanted to share what they were going through with someone who had lived that experience; one participant [1A12] recounted that the website could be used to create an online community of diabetes patients, who could act as a virtual peer support group (Table 2; Quote 2a).

They identified a desire to communicate with other patients, not just HCPs, to obtain health information. They identified other people with their disease as possible resources for health information, given shared life experiences, and that the website was one potential way to enable this (Table 2; Quote 2b). However, this desire to share experiences was tempered by a perceived need for privacy, with some participants describing how some individuals are not ready to share their experiences (Table 2; Quote 2c). These concerns for privacy must also be addressed in the online environment; we adopted “usernames” rather than the individual’s real name to preserve and respect their wish for privacy.

Despite the need to share and connect with others, participants indicated that it was a very individualized process, deciding with whom it was safe to share information and with whom it was not; they spoke of balancing the need for support with a need to be free from ‘scrutiny’ (Table 2; Quote 2d). Thus having an online forum in which to share experiences with others will likely suit some patients but will not meet the needs of others. A one-size-fits-all approach to fulfilling this ‘desire for community’ would not be appropriate.

#### Potential roles of an online self-management website in self-empowerment, behavior change, self-management and health care delivery

Participants identified the following potential roles of an online self-management website:

(a) *Motivation for behavior change*: Portions of the website gave rise to triggers for potential behavior change; for example, when viewing narrated videos and animations regarding diabetes care and prevention of complications, participants realized that diabetic morbidity could be reduced and recognized the value of self-care measures in preventing this morbidity (Table 2; Quote 3a). This balance between fear and hope engendered by the web tools emphasizes that when presenting potentially negative health information to people with diabetes, providing hope can be a potential enabler of positive behavior change.

   Similarly, participants found videos of patient testimonials provided “moral support” for ongoing behavior change: *“That was good. Like cheerleaders cheering you on. I think that one is very motivating. I like it when people get on it and talk.”* [3B12] Thus, the website, in particular video-based tools featuring both positive and negative aspects of diabetes and patient testimonials, could be viewed as motivating participants to try to change behavior.

(b) *Facilitation of self-monitoring and self-management tasks*:

The “tracker” functions and associated links to information were seen as tools to promote self-monitoring and stimulate next steps in self-management (Table 2; Quote 3b). One participant commented that use of the medication tracker would not only simplify his/her self-management tasks because a template was available, but also remind him/her regarding medication administration (Table 2; Quote 3c).

These findings contrast with other participants’ reports regarding the tediousness of self-management. Some participants expressed that self-management is onerous, as is recording one’s self-management efforts (Table 2, Quote 3d). While self-management can be a burden, the website may facilitate some of these tasks, and serve to offset this burden.

(c) *An adjunct to care between visits to HCPs*:

When discussing their information and emotional needs, participants spontaneously expressed frustration over their interactions with HCPs. Specifically, they described challenges with gathering information from physicians and highlighted that the time constraints characteristic of clinic visits were a concern. One participant noted, *“Your physician doesn’t tell you because he’s in such a big hurry to get you out of the office”* [2B06]. Thus, participants viewed their health care appointments as missed opportunities to gather important information (Table 2; Quote 3e). Such time-limited interaction with physicians was seen as constraining their ability to obtain knowledge/support for behavior change. As a result, several participants saw the website as complementary to medical care, emphasizing immediate availability and perceived credibility of HCPs who created the website as key factors impacting their use of the website (Table 2; Quote 3f). Moreover, participants felt that the website could be used in between visits with HCPs, addressing their need for “immediate” answers.

(d) *Facilitation of interactions with HCPs*: Several participants commented that some of the self-monitoring tools could allow them to present their self-monitoring data to their HCP as a way to optimize their limited appointment times: "(Table 2; Quote 3 g). Reports that they could print out from the website were seen as useful tools to present to their HCP in order to facilitate their care.

#### Importance of a patient-centered perspective in presenting content

Participants were sensitive to the tone and implications of website titles, formats, text and video, highlighting the importance of a patient-centered perspective when developing content. For example, when prompted to comment on the structure of the home page, which was designed to prompt users to select what they wanted (knowledge, skills, behavior change, reinforcement and support) in an effort to tailor to the individual, one user commented:

*“This title ‘behavior change’ is pretty presumptuous: it’s assuming I have to make the change. It’s kind of condescending.”* [2B01]

This participant’s comment suggests that attention should be devoted not only to language, but also to the assumptions underlying content. Offering insights as to why behavior changes might be advisable and framing the message in terms that are acceptable to patients is an important consideration in website design. If patients feel resistant to behavior change, the website can offer interactive risk assessment to explain the consequences of such decisions.

Participants also commented on the use of medical terminology within the website. Some participants critiqued such terminology.

*“I have a problem with the word ‘side effects’. They are not side effects, they are effects. […] If you take a medication and it really upsets you and [you] end up dropping to the can every 5 minutes, that’s an effect.”* [3B56]

This participant perceives the term ‘side effect’ as minimizing the impact that certain drugs can have on the individual. Rather than interpreting the term the way it is used in medical circles (as an unintended consequence of taking a drug), this participant demonstrates that the meaning is very different for the person experiencing it.

Many participants commented that they struggled to reconcile negatively perceived content with positive take-home messages. For example, the website included a video that graphically depicted the consequences of poor dental hygiene in diabetes, which concluded with an upbeat message that these consequences could be prevented by seeking timely medical care (Table 2; Quote 4a). This participant found the juxtaposition of such negative and positive messaging jarring and that the negative images might be enough to scare patients away.

Finally, participants highlighted the importance of tailoring content to the individual’s educational and cultural context (Table 2; Quote 4b). Thus, paying careful attention to common assumptions, medical nomenclature, language, messaging and sociocultural context can optimize the acceptability of the website to people with diabetes. The importance of considering the patient perspective is particularly amplified with web-based tools compared to print tools, given the range of media that are potentially employed with web-based tools (for example, video), as well as the multitude of options to organize the content (three-dimensional linkages in web-based tools, compared to print tools where organization is linear, and limited to only two-dimensional linkages)

#### Barriers and facilitators to use of a self-management website

Perceived lack of relevance of materials on other publicly available websites was seen as a barrier to their use; while participants were aware of online resources, they were disinclined to access them as they perceived that they would be of limited applicability to their specific situation (Table 2; Quote 5a). Ensuring the website was incorporated as part of their usual self-care and computer usage routine would facilitate its use by participants (Table 2; Quote 5b&c). Other facilitators of website uptake included the website’s availability for goal-directed use (Table 2; Quote 5d).

### Phase 3: identified usability issues

Usability testing revealed problems in multiple domains, including website layout and organization, website navigation, visual elements, data entry, interactivity, language, tracker layout and report layout (Table 3; a-h).

**Table 3:** Actions in response to findings from usability study

**Identified areas with usability problems**	**Solution implemented**
(a) Website layout and organization:	Reorganization of website categorization (from “type of tool” to “tool topic”), with provision of introductory information for website components, and intuitive overview of available content; specifically:
● Unclear scope, content and purpose of website	
	• Removal of knowledge, skills, behavior change, reinforcement and support categorization of tools
	• Inclusion of introductory page for first-time users
	• Inclusion of introductory page to sub-categories
	• Inclusion of a crumb trail
	• List of subtitles at top so viewers can get overall sense of the page
	• Combining Journal with Tracker functions
	• Grouping trackers all together in separate section of home page
	• Making log section more prominent
	• Description of purpose of logs
(b) Website navigation:	Simplification of search strategy, and presentation of search results, as well as providing intuitive links between recommended content
● Multiple search options confusing	• use only one search option (Boolean) and include examples
● Too many search results	• changed search algorithm, keyword system, sorted by relevance, show number of search results
● Presentation of search results overwhelming	• Indicate category of search result
	• Underline links, remove extraneous bullets (that look like buttons)
● Content not grouped in meaningful way	• Tool titles and descriptions to be more concise and skimmable, simplify wording
	• Alphabetical listing of topics, phrasing of title
	• Smart recommender widget
	• Links between related concept
(c) Visual elements:	Incorporation of icons, colors, bolded and larger font and diagrams; specifically:
● Limited use of meaningful aids, graphics, colors, fonts or alerts to help interpret data and facilitate learning	• Icons to differentiate interactive vs. non-interactive tools, Print function, Report function, Graph function, pill bottles
	• Highlight keywords
	• Increased color contrast
	• Inclusion more color within programs
	• Increased default font size
	• Ensure important content is visible without having to scroll
(d) Data entry:	Reduction of data entry tasks with automation of unnecessary tasks; specifically:
● Complex data entry tasks and unwanted workload	• Date/default info filled in automatically
	• Launch search automatically
	• Place cursor at beginning of each relevant field
	• Add a pull down menu for date and time, such as a “rolodex clock” for date and time
(e) Interactivity:	Incorporation of immediate feedback in response to user input; specifically:
● Limited attention-attracting features an feedback to engage user	• Immediate feedback after completing checklist (e.g. pop-ups to congratulate)
	• Provide feedback so user can check their “score”, e.g. ‘6 out of 10’
(f) Language:	Tailoring of content to lay-person; specifically:
● Information and instructions not suitable for users’ task and skill level	• Avoid medical terminology
	• Avoid abbreviations (FAQ, BP)
(g) Tracker layout	Clarification of actions required through use of buttons, clues, and alerts; specifically:
● Not intuitive in navigation and actions required to be taken	• Clarification of next steps, buttons more prominent
	• Inclusion of “clues” on how to enter in information (picture of prescription bottle label, with boxes and arrows indication which information is to be entered)
	• Making entries editable
	• Inclusion of tool that lists blood pressure readings and indicates when blood pressure is getting dangerously high
(h) Report layout	Provision of tailored report options relevant to the user’s needs; specifically:
● Display of information not tailored to user’s needs	• Inclusion of options to include summary or all readings
	• Inclusion of only necessary info
	• Incorporation of options to add physician’s names, phone numbers and locations.

### Phase 4: intervention refinement

Based on feasibility and usability testing sessions, we made revisions to the website in an iterative fashion after each cycle of testing, over an 8-month period. Figure 3b depicts a screenshot of the home page highlighting changes made based on user evaluation. Additional file 1: S4 provides representative screenshots of a topic page “Blood sugar”, a sample tracker “Medication Log”, the blog, sample peer story-telling and sample interactive goal-setting “My profile”.

(a) Actions in response to findings from the feasibility study:

In order to address *the desire for control* in the online setting, we wanted to include tools that permitted patients to obtain tailored information suited to their needs, and to direct their own care. Specifically, we included a blog, with invitation to comment, share and ask questions, twice weekly, as well as an “ask the expert” topic, consisting of blog postings from health care providers such as endocrinologist, pharmacist and dietitian (Additional file 1: S4c). We also provided a selection of recommended pages based on previous pages used and user-specific data (bottom of Additional file 1: S4e). Finally, we included tools to help patients prepare for health care provider appointments to direct their own care (such as tip sheets and reports).

To address *the desire for community* in an online setting, we sought to develop an online social networking community in which they could ask and answer questions, share concerns, and provide encouragement. Thus, we included an open forum on which participants could post and respond to others’ comments (Additional file 1: S4c).

In order to *motivate for behavior change,* we included evidence-based behavior change strategies such as interactivity (Additional file 1: S4b,e), goal-setting (Additional file 1: S4e), feedback (Additional file 1: S4b) and peer story-telling (Additional file 1: S4d). In addition, we incorporated evidence-based resources, monitoring tools (for example, medication trackers, Additional file 1: S4b) and instructional videos to facilitate self-management (Additional file 1: S4d)*.* Features to enhance patient-physician communication (for example, how to prepare for appointment, self-management reports) were created to fulfill the role of *being an adjunct to care between visits to HCPs*.

We addressed the importance of *a patient-centered perspective* by reviewing and rephrasing the content. For example we avoided the term “behavior change”, and edited the text to ensure an appropriate reading level. We also reorganized the structure of the website as indicated in Table 3; a-h.

To reduce *barriers to website use*, namely perceived lack of relevance and not being part of the usual routine, we revised the home page layout such that topics and resources of particular interest and relevance (such as trackers) were immediately visible (Figure 3b). We also included an introductory statement for each tool, emphasizing features of reported relevance and utility. We addressed identified usability issues to minimize disturbance to usual routine (Table 3; a-h). To leverage *facilitators of website use*, such as its availability for goal-directed use, we ensured ease of navigation as indicated in Table 3 and included links to related concept and a side-bar of recent tools (Figure 3b).

(b) Actions in response to findings from the usability study:

We addressed each of the usability problems identified in Table 3; a-h. For example, to address problems in website layout and organization, we reorganized website categorization (from “type of tool” to “tool topic”) and provided introductory information for website components as well as concise overviews of available content; specific details are included in Table 3; a-h.

## Discussion

Our study confirmed previously reported findings, but also revealed unexpected insights regarding the informational needs of individuals with type 2 diabetes, providing us with important feedback to inform the development of our website. While our findings confirm the existing literature, we also demonstrate their continued relevance in today’s digital age and applicability to web-based interventions. Specifically, our findings support and highlight the relevance of self-efficacy as the theoretical platform of our online intervention, demonstrating its applicability to web-based media. Participants discussed the potential utility of self-monitoring and reflection with the trackers *(successes or failures during previous performances)*, looked to peers’ experiences in video testimonials and the blog *(observations of others’ experiences)*, selected the amount and content of information that fit their acceptable worldview *(selective processes),* identified the website as a motivator of behavior change *(motivational process)*, and demonstrated visceral emotional responses to some website content *(physical and affective processes)*[18].

Our findings regarding patients’ struggles with self-management in the modern-day, online context reflect pivotal findings in chronic disease. We found that patients’ traditional struggles with self-management apply also to the online context. Specifically, our patients’ accounts of the tediousness of self-management in the online context reflect Corbin and Strauss’ “illness work” [40]. Similarly, the disruption of web-based self-management into daily life, one of the identified barriers in our study, is echoed in their concept that work, including “illness work” and “everyday life work” that must be sequenced and fit into each other. Thus our findings build upon previous understandings of chronic disease management, as applied to newer technologies.

Our study also confirmed the complexity of informational needs and resources that individuals with diabetes seek [19]. In the context of developing a self-management intervention rather than a peer support community, we unexpectedly found the need for a personalized and supportive environment including emotional support for patients with diabetes. A survey of 1159 patients recently diagnosed with diabetes found variability in patient needs for emotional support; 23% of respondents wanted more emotional support [41]. Similarly, a systematic review of online weight management programs underlined the importance of recreating the human experience and of providing a supportive experience as key principles in the development of web-based programming [42]. Despite these findings, a systematic review of the impact of online social network interventions on health behavior identified 10 studies of diet, physical activity and/or weight loss interventions in healthy, overweight adults and cancer survivors, and found that effect sizes were variable but generally small and statistically non-significant [43]. Authors concluded that this area is still in its infancy, requiring optimization of these interventions in order to achieve sustained behavior change.

Our usability study highlighted the importance of an easy-to-use interface to maximize perceived relevance and thus continued use of the online self-management website. These findings echo the conclusions of a systematic review of electronic resources for diabetes self-management in the published and grey literature [11]; this systematic review concluded that while a large number of studies and tools were identified in the search, only 57 studies assessed outcome and 60% of these had 3 or more usability problems. The systematic review also identified that interactivity and feedback may play a role in persistent website use, which may be associated with greater improvement in patient outcomes [11], features that we had also incorporated based on findings of the current study. Our usability findings and revisions regarding website navigation, visual elements, data entry, interactivity and language are applicable to many other health website designers; while our findings regarding website, tracker and report layout are specific to our system, similar features can be transferred to other chronic disease websites (for example, respiratory symptom tracker for asthma, summary report of daily function for rheumatoid arthritis). Our findings will guide developers who wish to incorporate these features into their system.

Strengths of our approach include our rigorous theory-driven and evidence-based approach to intervention development, our systematic refinement of the intervention based on feasibility and usability data, and our selection of usability assessment techniques. Limitations of our feasibility study include a small sample size of participants recruited from a single city. However, we had a heterogeneous sample with a range of characteristics and experiences of the target population of patients living with diabetes with access to the internet and we were able to acquire a rich data set that was used to assist in the refinement of the tools. This suggests that these findings will be transferable beyond the study setting. In addition, we followed rigorous qualitative methodology, by using trained moderators who were not otherwise invested in the project, and employing independent coding by two individuals and interpretation by three individuals to ensure data trustworthiness [35]. Additional methods for ensuring analytic rigor include our use of constant comparative analysis to explore similarities and differences of participants’ experiences and to ensure that iterations of interview guides reflected emerging analysis [44]. Furthermore, we had prolonged and intensive engagement with participants across the entire study as a technique for promoting trustworthiness of our analysis [44,45].

## Conclusion

In our study, participants expressed a desire for control and for community, through greater access to timely and personalized knowledge, support and health care. They viewed the website as a motivator for behavior change, a facilitator of self-monitoring, an adjunct to health care and a facilitator of HCP visits. They also highlighted the importance of patient-centered approaches to information-sharing and identified mediators to website use. Findings from our usability testing confirmed the need for interactivity and easy-to-find answers to participants’ questions about their diabetes care. We addressed these findings by including a forum and blog and tools to help patients prepare for appointments (such as a pre-appointment checklist and printable reports), revising website layout and navigation, selecting recommended pages based on user-specific data, addressing usability issues to minimize disturbance to usual routine, and providing information they deemed valuable on our website (Table 3, Additional file 1: S3).

The first four phases of this five-phase study have shed light on information needs of patients with type 2 diabetes. The last phase examines how and why participants used the website, and its impact on important clinical and psychological outcomes and is the focus of a forthcoming publication.

## Competing interests

The authors declare that they have no competing interests.

## Authors’ contributions

CY conceived of the study, analyzed the data, led the discussions and drafted the manuscript; she is guarantor of the manuscript. JAP participated in the study design, analyzed the qualitative data and contributed to the discussion. SH facilitated the sessions and analyzed the qualitative data. AJ and DL conducted the focus group and usability sessions. BRS facilitated study conduct and contributed to the discussion. SES participated in the design of the study, contributed to the discussion. All authors revised the manuscript critically for intellectual content, and have read and approved the final manuscript.

## Pre-publication history

The pre-publication history for this paper can be accessed here:

http://www.biomedcentral.com/1472-6947/14/60/prepub

## Supplementary Material

Additional file 1 Online supplementary material. Click here for file
