# Supplementary material for: User-centered design of a web-based self-management site for individuals with type 2 diabetes – providing a sense of control and community
Source: BMC Med Inform Decis Mak. 2014 Jul 23;14:60. doi: 10.1186/1472-6947-14-60 (PMC4222757; doi:10.1186/1472-6947-14-60)
Supplement: Additional file 1 — Online supplementary material. [file 1472-6947-14-60-S1.pdf]

Online Only Supplementary Material
Supplementary Appendix S1: Feasibility study interview guide

On behalf of the research team, I'd like to thank you all for coming in today. As you may know, the Ontario government is focusing their efforts on people with diabetes. As part of this effort, we have developed a few tools to help people with diabetes, with input from other people with diabetes. The purpose of this focus group is to get your opinion about these tools, so that we can better refine these tools. So thank you very much for agreeing to participate in this discussion.

(*Ask each person to introduce herself or himself.)*

GROUND RULES

The way this discussion will work is that I will start with some questions that I would like you to respond to. Please keep in mind that there are no right or wrong answers - we just want to get some ideas about your opinions and experiences. There should be some time for open discussion at the end.

If you have a different opinion than someone else, please feel free to say so. All I ask is that you give everyone a chance to state their view - and it helps if only one person is speaking at a time. Because I have a number of topics I want you to cover, I may have to ask the person speaking to cut short their answer. Please don't be offended; it's just that I may need to keep things moving so we can finish on time.

I'd like to remind you that everything you say will be kept strictly confidential. And I think you understand that our discussion will be audio taped because it is difficult to write notes and follow the discussion at the same time. *(Have participants complete and hand in informed consent forms at this point. Tell them about refreshments, need to turn tape over, and when we should end.)*

Are there any questions before we begin?

The laptop in front you has the browser open to the website that we will be looking at today. There is a sheet of paper beside you with the following task:

- You are wondering a) what your risk of heart disease is and
- b) how you might reduce this.

Please use this website to find the answer to this question, or as close an answer as you can find. Feel free to use the sheet of paper to take notes of comments, or of anything that surprises you or confuses. Your experience of what works and what is confusing will help guide us to improve the website. As you will see, we are looking at a prototype of the website, that we will complete based on your feedback today. As a result, you will note that at this point in development, not all pages will be available. However, those required for the task at hand are available.

(Allow participants 8-10 minutes or until everyone appears satisfied.)

The diabetes tools

1) Please comment on your experience? Where you able to find an answer? *(Get feedback from everyone.)*

2) Now I'd like to get your sense of some of the specific strengths and weaknesses of these webpages, so let's start with the strengths. What do you like about them, what are its good points, what works (in it)?
3) What are the weaknesses in these diabetes webpages? What doesn't work, in your view?
4) What content did you find particularly helpful?
5) What content did you find was not helpful?
6) Which aspects of the format did you find particularly helpful?
7) Which aspects of the format did you find not helpful?
8) If you could change just one thing about these tools, what would it be? *(Get feedback from everyone.)*

Regarding Website Content:

9. Feedback regarding Title: "Diabetes Knowledge Refinery" What imagery comes to mind, what connotation, what is your understanding

10. Complications: When thinking about the complications of diabetes (heart disease… foot care), what is most important to them? Preventing vs managing the complications?

Regarding Website Process:

11. What factors would impact your decision to visit this website for the first time? What would help/enable you to visit this website for the first time? What about subsequent visits: what factors would impact your decision to visit this website repeatedly? What would help/enable you to visit this website?

12. Some individuals with diabetes want an opportunity to share their experiences and for social support. This is called social networking. Some of you may have heard of facebook? This is an example of social networking. There are different forms of social networking. For example, a blog is when one individual (can be a person with diabetes, a diabetes educator etc) posts a comment, and other individuals can reply or respond to it. A forum is a bit of a free-for-all – anyone can post anything and respond to anything. Blog vs forum; which do they prefer and why? Which would they most likely use and why? Which is more comfortable and why? What features are valuable to you and why? Regarding moderation, what is your preference and why (none, person with diabetes, health care provider, layperson)? How do you see yourself using this resource and why – do you see yourself reading the posts, adding your own post? What factors affect whether or not you would post something?

13. Entering information: How do you feel about the website "remembering" data that you put in already, so that it is available for you when you use the next tool, or next time you use it? How do you feel about having the option to login in order to achieve above? What about entering in minimal anonymized information about yourself to help us assess website use?

Conclusion

14. We're almost finished with our discussion now but I'd like to give each of you one last chance to comment on this website. Your feedback in this evaluation has been extremely helpful but if you had to make one last recommendation regarding this website, whether it be something that has not been discussed yet or a reiteration of an earlier point, what would be your final word? *(Get feedback from everyone.)*

Thank participants.

Online Only Supplementary Material
Supplementary Appendix S2: Usability Testing Interview Guide

[Hand out consent form.]

Thanks again for taking the time to participate in our usability testing. It will probably take us about 60 minutes to complete. That may vary depending upon questions, interruptions, you know....

Before we get started I wanted to cover a couple of points.

First off, I wanted to ask your permission to video record the session. This will allow us to complete the interview **much faster** freeing me up to observe instead of madly writing notes.

Next, this phase of the project is focused on collecting feedback from potential users like yourself. The feedback we are looking for is from **you**. There is no right or wrong answers. It's not a test. That's important to keep in mind. We are interested in what you think about what is shown—the good, the bad, the confusing; we would like to know what you think.

That means I'm going to try to say as little as possible during the interview. That's why I actually have this script. Hopefully, it will keep me on track and my talking to a minimum. Using it (script) can sound a bit formal and bit awkward but the process calls for it.

The way this will work is that I will ask a set of prepared questions. If you feel like asking me a question to clarify something please go ahead. Depending on the question I might say: "***I will address thatat the end of the interview***"

Please do not be offended by this as I mentionedwe need to get your feedback, even if that feedback is a sense of confusion.

Any questions before we get started?OK, I'm going to turn on the recorder. Let's start.

[Hand out background questionnaire]

Diabetes is a complex disease that affects many aspects of your life. Because of this, a toolkit might help people with diabetes learn about diabetes and help them take care of themselves and live with diabetes. The purpose of these webpages and tools we are looking at today is to help people who have diabetes understand what diabetes is, and how to manage it. We need your help in order to improve this resource so that it can be useful for other people with diabetes.

THINK ALOUD INSTRUCTIONS
While you are using the website, we are interested to know what you're thinking, a method called 'Think Aloud'. What we mean by think aloud is that we would like for you say everything that you're thinking. In other words, you will be constantly talking, telling us what you are thinking inside your head.

For example, if I were to think aloud while trying to find the sixth letter of the alphabet, I would say all the letters and use my fingers to count a b c d e f there I found it.

Online Only Supplementary Material
Supplementary Appendix S2: Usability Testing Interview Guide

[Facilitator: Note down behaviours related to: Exploration, Task, Conceptual Model, Usefulness, Usability. Hand out tasks on a sheet of paper.]

Scenario 1: Imagine that you did not know about this website before, and that you do not know that it is being created by healthcare professionals. Imagine that You have just come across this website. You want to know how reliable this website is.

(a) Find out how reliable the information on the web site is.

Scenario 2: Imagine that you are concerned about your blood pressure. Please use this website to find an answer to the following questions. Please remember to think aloud as you are looking for the answer to each question.

a) What is blood pressure?
b) In what ways will having high blood pressure affect me?
c) What are concrete things that I can do to control my blood pressure?
d) You checked your blood pressure at the pharmacy right after a stressful day at work. It is 143/82. You want to keep a record of this using the website.

Scenario 3: You would like to enter your medications into your Diabetes Online Companion. As you do this, please remember to think aloud about what is working, what is easy, what is hard.

Scenario 4: You have been wondering in what formats the information about diabetes is available on this website. Specifically, a comic would be interesting.

a) Find a comic related to diabetes.

Scenario 5: Please read the two comic strips on the screen.

Comic1

a) What is your interpretation of this comic? What was the message?
b) What are your thoughts on these comics? Are they useful to you personally? If yes – why? Do you think they would be useful to other people with diabetes?
c) How did these comics make you feel? What did you like about them? What you didn't like? How would you make them better?

Comic 2

Repeat above questions.

d) What did you think about the navigation? Did you have trouble following it? Can you think of easier ways to go from one image to the next?
e) Some people associate "comic" to children and may not be interested in reading this. Does "graphic novel" appeal to you more than "comic"? If you were not asked to check this during this session, would you be interested in reading this on your own?
f) How does reading this comic compare to reading information about the same topic? How does it compare to sharing experiences with other individuals with diabetes?
g) These comics were written to allow people to see or share someone else's experiences with diabetes. Did you have that experience? If not, why not? What can we change?

Scenario 6: You've forgotten what your blood pressure was and you want to look it up.

a) Find out what your blood pressure was today.

Online Only Supplementary Material
Supplementary Appendix S2: Usability Testing Interview Guide

After each scenario, ask the following question:
Please comment on your experience? Where you able to find an answer?

Final questions after scenarios are complete:

9) Now I'd like to get your sense of some of the specific strengths and weaknesses of these webpages, so let's start with the strengths. What do you like about them, what are its good points, what works (in it)?
10) What are the weaknesses in these diabetes webpages? What doesn't work, in your view?
11) What content did you find particularly helpful?
12) What content did you find was not helpful?
13) Is there any missing content to the website?
14) Which aspects of the format did you find particularly helpful?
15) Which aspects of the format did you find not helpful?
16) If you could change just one thing about these tools, what would it be?
17) Did you notice the evidence levels? Are they useful to you?
18) Entering information: How do you feel about the website "remembering" data that you put in already, so that it is available for you when you use the next tool, or next time you use it? How do you feel about having the option to login in order to achieve above? What about entering in minimal anonymized information about yourself to help us assess website use?
19) Please let us know any final comments that you have on anything good, bad or confusing that will enable to identify problems and improve the website.

Thanks for your participation.

Online Only Supplementary Material
SupplementaryAppendix S3: Additional content requested by usability participants

Medication	Information on drug interactions
	Instructions, e.g. how to take insulin
	List of common medications and categories of medications (e.g. medications with different names), as well as how these medications function.
	Importance of taking the medications exactly as prescribed. For example, why some medications should be taken with food.
	"How-to" information on insulin
Food & Alcohol	Information on interpreting food labels, glycemic indices of foods
	More information on why alcohol intake is of concern to diabetics
Other	Information on healthy waist ranges
Comics	Topics on ways to combat stress, such as meditation.

Online Only Supplementary Material

Supplementary Appendix S4: Representative screenshots from the Diabetes Online Companion

a) Screenshot of Sample Topic Page "Blood sugar"

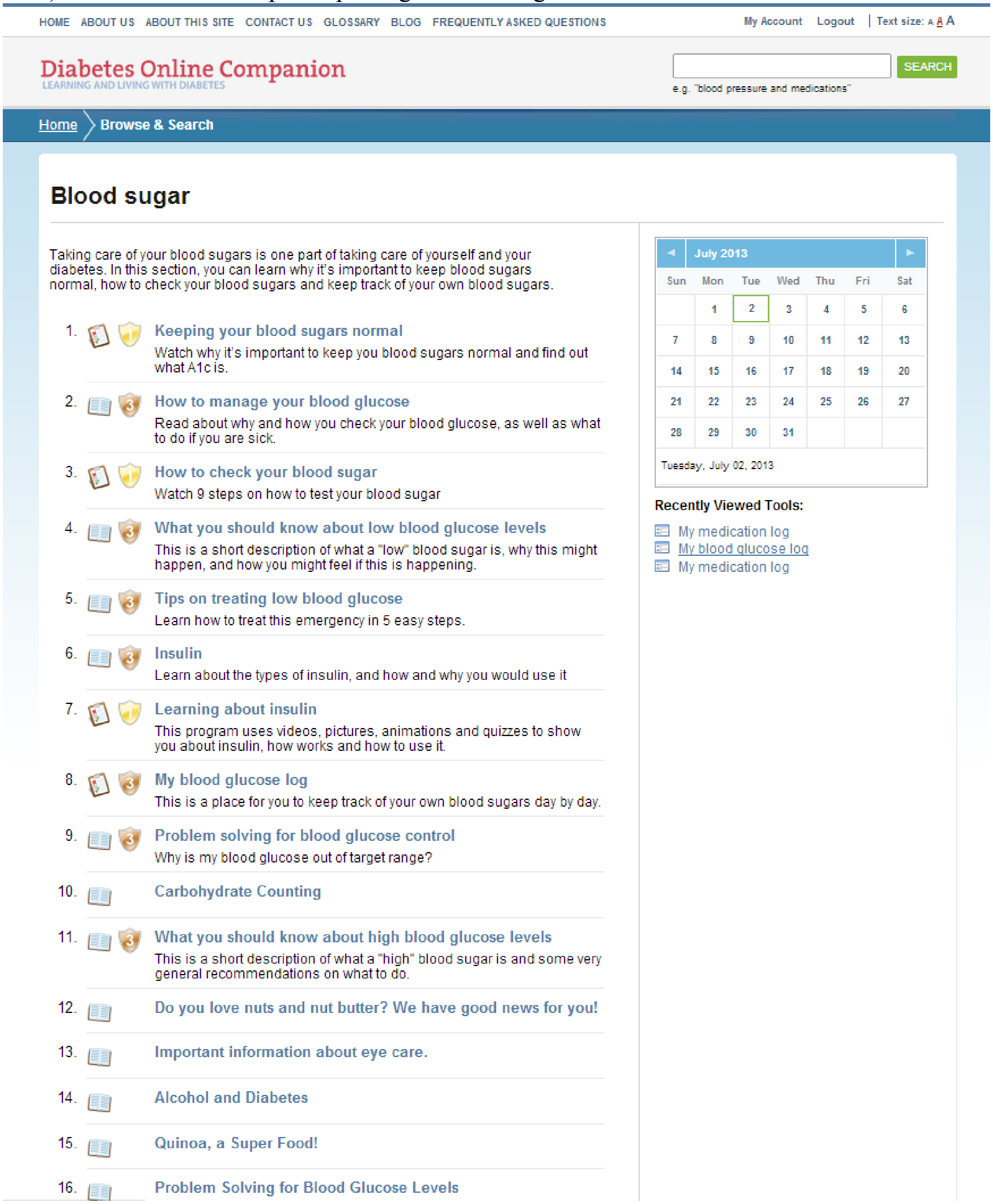

Online Only Supplementary Material
Supplementary Appendix S4: Representative screenshots from the Diabetes Online Companion

b) Screenshot of Sample Tracker "Medication Log":

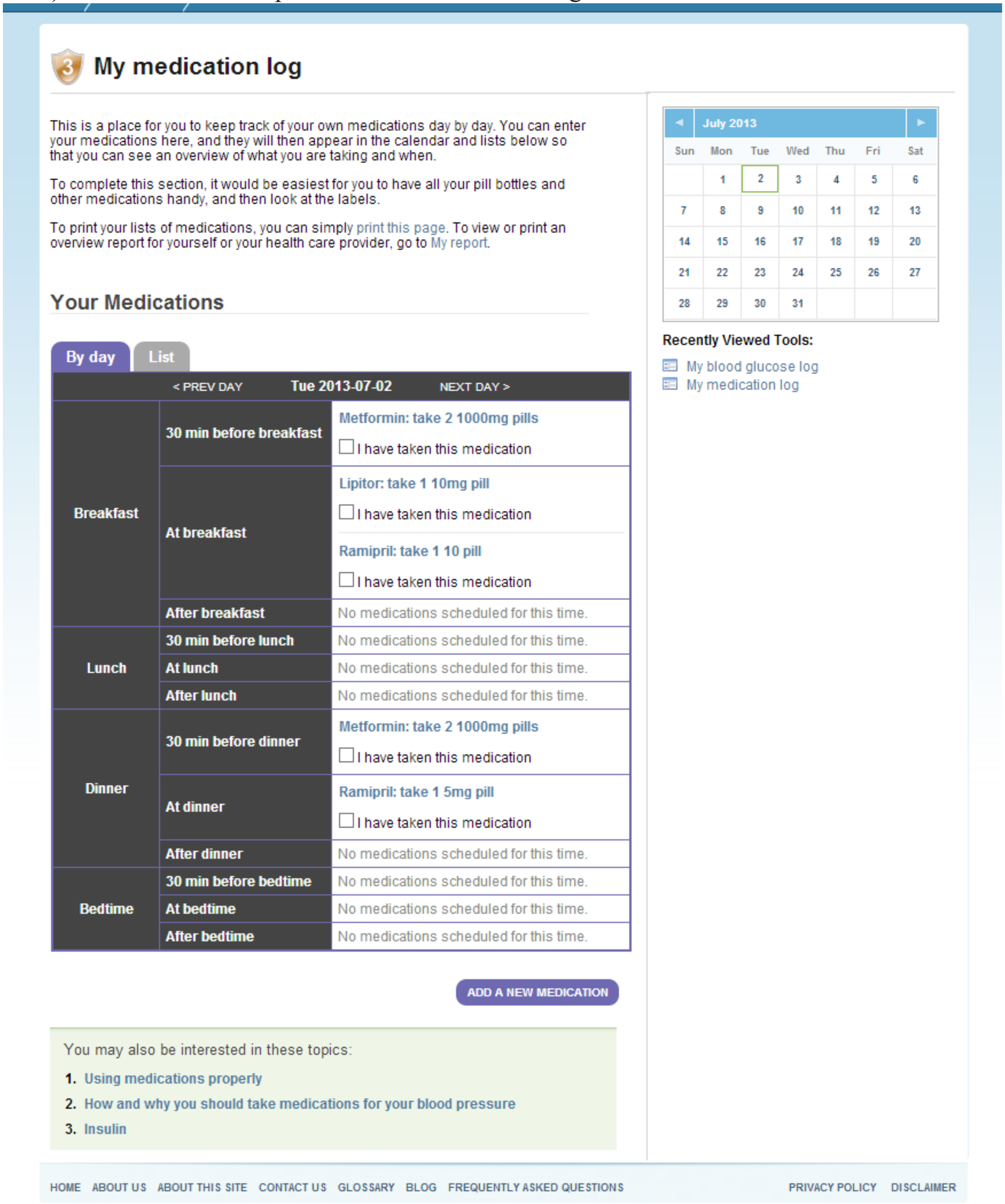

Online Only Supplementary Material

Supplementary Appendix S4: Representative screenshots from the Diabetes Online Companion

c) Sample blog:

Blog

Toggle Comment Threads

Your Diabetes Online Companion (Reviewed by an Endocrinologist)
7:00 am on November 16, 2011
Permalink | Reply | Edit

Tags: Eye care (2), Foot Care (2), glycemic index (8), Share (4), Support (3)

Ask the Expert!

We are offering another opportunity to ***"Ask the Expert"***.

Don't limit your questions to something that you would ask only a physician.

Think about what you might want to ask a dietician or an ophthalmologist or a podiatrist.

Maybe you would like to know more about the **glycemic index**. Did you know that Dr. David Jenkins, who pioneered the research behind the glycemic index, is a physician and researcher at St. Michael's?

You have a wealth of information right here, at your fingertips. Don't be afraid to ask **ANY** questions related to diabetes that you might have.

Remember, your posts are anonymous and you can bet that someone else has been asking themselves the same question that you post….you will be helping them as well.

Your Diabetes Online Companion (Reviewed by an Endocrinologist)
12:20 pm on November 3, 2011
Permalink | Reply | Edit

Tags: Blood pressure (10), Medication (16)

Medications Affecting Blood Pressure

Besides medications intended to treat your heart disease and high blood pressure, other medications can affect blood pressure. These include such things as both prescription and over-the-counter non-steroidal anti-inflammatory medications (NSAIDs) used to treat pain and injuries with inflammation – the NSAID family includes medications such as ibuprofen (Advil), naproxen (Naprosyn), sodium naproxen (Aleve), and indomethacin (Indocid). NSAIDs can cause your kidney to retain salt and fluid in the body, which ultimately raises the blood pressure.

Caffeine can also raise your blood pressure (at least for the short-term) – caffeine does this by causing your heart to beat more often, which means that more blood is pumped around the body thereby raising your blood pressure. Remember to moderate your caffeine intake. Heavy use of alcohol may also increase your blood pressure. Oral decongestant medication (frequently contained in many cough and cold medications) may increase blood pressure as well, so you need to be careful about which cough and cold meds you use.

Some prescription medications such as oral contraceptive pills can increase blood pressure as well and you will need to be followed closely for this if you are taking such pills. Conversely, some prescription medications can lower [illegible]

Archives

November 2011
October 2011
September 2011
August 2011
July 2011
June 2011
May 2011
April 2011
March 2011
February 2011
January 2011

Recent Posts

Ask the Expert!
Medications Affecting Blood Pressure
Ask the Expert!
Makeover your favourite family recipes
Eating Away From Home

Recent Comments

Your Diabetes Online Companion (Reviewed by an Endocrinologist) on Ask the Expert!

1A06 on Ask the Expert!

1A06 on Ask the Expert!

3B53 on Ask the Expert!

Your Diabetes Online Companion (Reviewed by an Endocrinologist) on Ask the Expert!

Online Only Supplementary Material
Supplementary Appendix S4: Representative screenshots from the Diabetes Online Companion

d) Sample peer story-telling "How do you prevent kidney damage"*

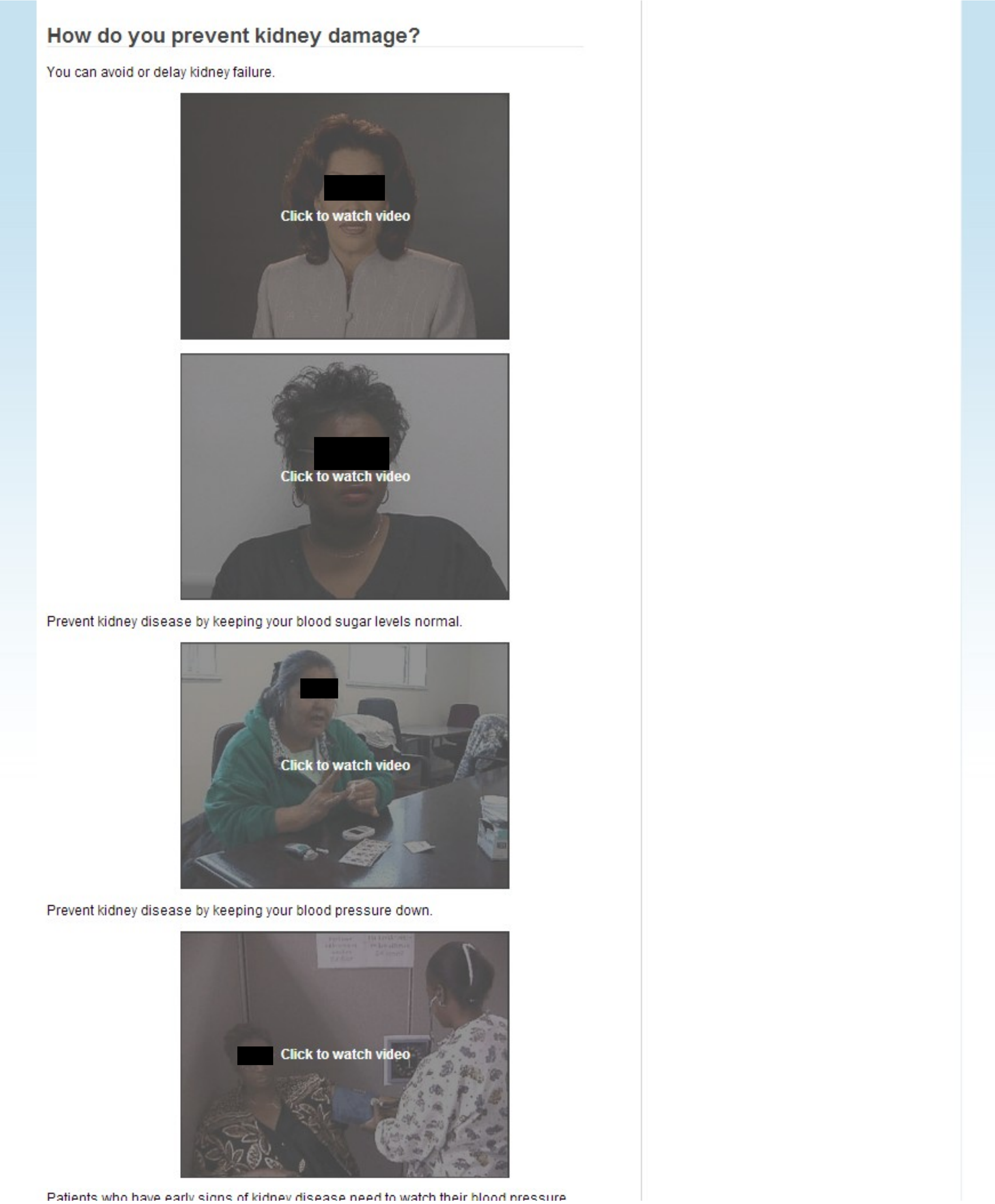

*Videos adapted from Living Well with Diabetes , University of Illinois at Chicago, represented here with permission (Gerber BS, Brodsky IG, Lawless KA, *et al*: Implementation and evaluation of a low-literacy diabetes education computer multimedia application.*Diabetes Care* 2005, 28:1574–80.)

Online Only Supplementary Material

Supplementary Appendix S4: Representative screenshots from the Diabetes Online Companion

e) Sample interactive goal-setting "My profile":

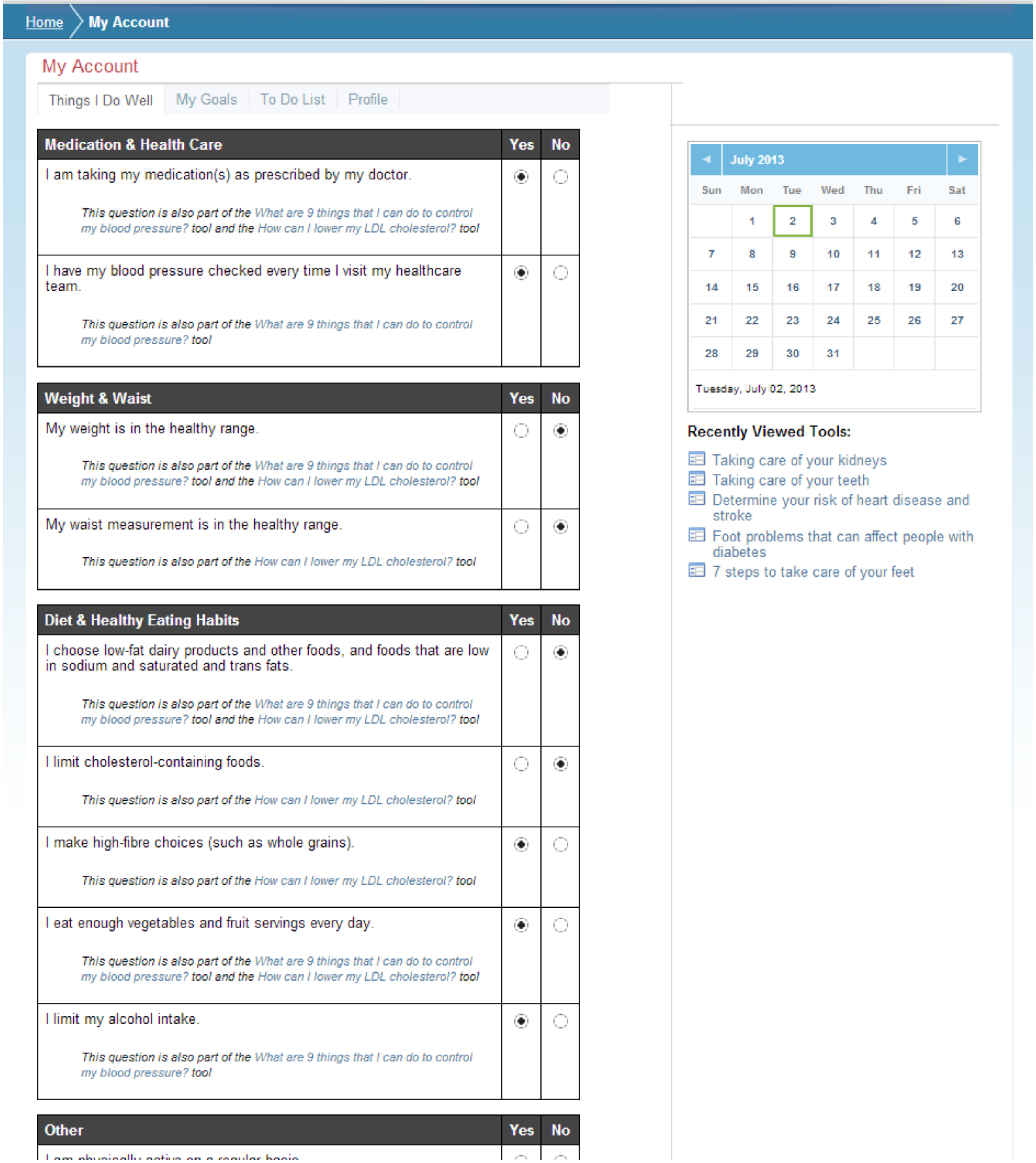

Home > My Account

My Account

Things I Do Well | My Goals | To Do List | Profile

Medication & Health Care	Yes	No
I am taking my medication(s) as prescribed by my doctor. *This question is also part of the What are 9 things that I can do to control my blood pressure? tool and the How can I lower my LDL cholesterol? tool*	●	○
I have my blood pressure checked every time I visit my healthcare team. *This question is also part of the What are 9 things that I can do to control my blood pressure? tool*	●	○

Weight & Waist	Yes	No
My weight is in the healthy range. *This question is also part of the What are 9 things that I can do to control my blood pressure? tool and the How can I lower my LDL cholesterol? tool*	○	●
My waist measurement is in the healthy range. *This question is also part of the How can I lower my LDL cholesterol? tool*	○	●

Diet & Healthy Eating Habits	Yes	No
I choose low-fat dairy products and other foods, and foods that are low in sodium and saturated and trans fats. *This question is also part of the What are 9 things that I can do to control my blood pressure? tool and the How can I lower my LDL cholesterol? tool*	○	●
I limit cholesterol-containing foods. *This question is also part of the How can I lower my LDL cholesterol? tool*	○	●
I make high-fibre choices (such as whole grains). *This question is also part of the How can I lower my LDL cholesterol? tool*	●	○
I eat enough vegetables and fruit servings every day. *This question is also part of the What are 9 things that I can do to control my blood pressure? tool and the How can I lower my LDL cholesterol? tool*	●	○
I limit my alcohol intake. *This question is also part of the What are 9 things that I can do to control my blood pressure? tool*	●	○

Other	Yes	No
I am physically active on a regular basis	[illegible]	[illegible]

July 2013

Sun	Mon	Tue	Wed	Thu	Fri	Sat
	1	2	3	4	5	6
7	8	9	10	11	12	13
14	15	16	17	18	19	20
21	22	23	24	25	26	27
28	29	30	31			

Tuesday, July 02, 2013

Recently Viewed Tools:

- Taking care of your kidneys
- Taking care of your teeth
- Determine your risk of heart disease and stroke
- Foot problems that can affect people with diabetes
- 7 steps to take care of your feet
